# Use of an ultrasound picture archiving and communication system to answer research questions: Description of data cleaning methods

**DOI:** 10.1002/ajum.12374

**Published:** 2024-01-13

**Authors:** Matthew K Moore, Gillian Whalley, Gregory T Jones, Sean Coffey

**Affiliations:** ^1^ Department of Medicine HeartOtago, Dunedin School of Medicine, University of Otago Dunedin New Zealand; ^2^ Department of Surgical Sciences, Dunedin School of Medicine University of Otago Dunedin New Zealand; ^3^ Department of Cardiology Dunedin Hospital, Te Whatu Ora Southern Dunedin New Zealand

**Keywords:** data cleaning, data linkage, echocardiography

## Abstract

**Introduction/Purpose:**

Ultrasound picture archiving and communication system (PACS) databases are useful for quality improvement and clinical research but frequently contain free text that is not easily readable. Here, we present a method to extract and clean a semi‐structured echocardiography (cardiac ultrasound) PACS database.

**Methods:**

Echocardiography studies between 1 January 2010 and 31 December 2018 were extracted using a data mining tool. Numeric variables were recoded with extreme values excluded. Analysis of free text, including descriptions of the heart valves and right and left ventricular size and function, was performed using a rule‐based system. Different levels of free text variables were initially identified using commonly used phrases and then iteratively developed. Randomly selected sets of 100 studies were compared to the electronic health record to validate the data cleaning process.

**Results:**

The data validation step was performed three times in total, with Cohen's kappa ranging between 0.88 and 1.00 for the final set of data validation across all measures.

**Conclusion:**

Free text cleaning of semi‐structured PACS databases is possible using freely available open‐source software. The accuracy of this method is high, and the resulting dataset can be linked to administrative data to answer research questions. We present a method that could be used to answer clinical questions or to develop quality improvement initiatives.

## Background

Ultrasound is central to modern medical practice, with ongoing increases in clinical use seen in recent years.^1^, ^2^ In addition to the clinical report, local picture archiving and communication system (PACS) databases contain rich data, which can be used for quality improvement and clinical research. However, these databases frequently contain multiple free text fields, making analysis challenging, such that studies using PACS databases often only use numeric or clearly defined categorical variables.

A number of recent studies have highlighted the value of such studies. A research group in Massachusetts, USA, has successfully examined the surveillance of mitral valve disease, by using a local database of echocardiograms with structured, granular clinical data, between 2001 and 2016.^3^ Another team in Israel has linked their structured echocardiogram report database to nationally collected mortality data, to examine several valvular abnormalities.^4^, ^5^ Work in Australia has resulted in the National Echocardiographic Database Australia (NEDA), which has already been used to look at mortality in those with moderate aortic stenosis and pulmonary hypertension.^6^, ^7^, ^8^

However, semi‐structured or unstructured PACS databases can make the conversion of qualitative variables into usable data difficult, which may discourage researchers from doing so. Frequently, results from such studies are presented without providing sufficient information on the extensive data cleaning (especially of free text data) and validation processes to allow other investigators the ability to implement such processes in their local database. In this paper, we present a methodology for cleaning a semi‐structured echocardiography database and hope that the methods described here could be used by other researchers interested in examining their own ultrasound databases.

## Methods and results

### Setting and participants

The Southern District Health Board (SDHB, now named Te Whatu Ora Southern) provides tertiary cardiology services to the lower part of the South Island, New Zealand, with approximately 5000–6500 echocardiograms performed annually for the approximately 330,000 population. The study cohort comprised all patients who had an echocardiogram at our local tertiary cardiology hospital between 1 January 2010 and 31 December 2018.

### Echocardiography report extraction

The echocardiographic PACS used in SDHB is Syngo Dynamics (version VA20F; Siemens Healthineers, Erlangen, Germany). Data were extracted using the proprietary Syngo Dynamics Data Miner, and output as a comma‐separated values (csv) file. There were several hundred variables accessible to the data miner, and the vast majority of these were not in use. Individual variable names differ between different versions of the clinical echocardiography report, and so manual identification is needed for extraction. We wished to extract a set of variables that would provide the relevant information about the aortic valve, alongside key markers of heart function. For brevity, these are included in the Supplementary Material (Table S1).

The data miner can save the search as a .xml file, allowing for reproducibility. In short, the data miner extracted all studies between 1 January 2010 and 31 December 2018 that were tagged as an echocardiogram. The specific variable names within the PACS can be found in this .xml, alongside a list of the final echocardiography variables these were used to generate. Point of care ultrasound studies are not archived in our local system and hence would not be extracted via this method. In total, 42,517 echocardiography studies were extracted.

#### De‐identification

Data were de‐identified in the following way:

1. A random 10‐character identifier (the ‘Anonymous ID’) was generated for each unique National Health Index (NHI) number. Each de‐identified ID was linked to an NHI number in a lookup table.
2. Using the lookup table, NHIs in the main dataset were replaced with the anonymous ID.
3. The lookup table was then saved and stored securely in accordance with the study protocol, separate from the de‐identified data that were used for analysis.

### Echocardiography report variable cleaning

The data miner was unable to extract all studies into a single .csv file, due to computational power constraints. Hence, extracted studies were stitched together into a single dataset. All data cleaning and linkage were performed using R version 3.6.3^9^, RStudio version 2021.09.01^10^ and the R package ‘tidyverse’.^11^ The NA designation in variable refers to not available and labels missing data.

The order of data cleaning is summarised in Table 1.

**Table 1:** Order of data cleaning.

Data cleaning step	Variables cleaned
Data loading	Data loaded into R, stitched together and anonymised
Renaming	Variables renamed for ease of cleaning
Remove unused variables	Variables where every observation is NA were identified and excluded
Study date, age, sex, height, weight and BMI	Study date, patient age and patient sex reformatted. Height and weight formatted, with manual correction to erroneous entries. Body mass index calculated, with a BMI ≥ 70 kg/m^2^ or ≤14 kg/m^2^ excluded as out of plausible range
Indication free text analysis	Free text analysis to identify indications
Cardiac rhythm and heart rate	Free text analysis to identify cardiac rhythm and formatting of heart rate (see below). Heart rate values above 200 beats per minute were excluded
Left ventricle numeric variables	IVSd, LVPWd, LVEF and LVOT diameter systole reformatted with impossible values (≤0) removed
Left ventricle and atrium free text variables	Free text analysis to identify left ventricular size and function, alongside left atrial size
Mitral valve	Mitral E, A, e′, E/a, and E/e′ reformatted with impossible values (≤0) removed. Free text analysis to identify mitral stenosis, regurgitation, annular calcification and prolapse. The presence of annular calcification or stenosis was then used to create a binary variable ‘any mitral calcification’
AoV	Ascending aorta diameter, aortic root diameter, AoV Vmax, AoV mean pressure gradient, AoV VTI and AoV area reformatted with impossible values (≤0) removed. Aortic valve dimensionless index was calculated as LVOT VTI divided by AoV VTI. Free text analysis to identify AoV stenosis, AoV regurgitation and AoV morphology/structure
Pulmonary valve	Free text analysis to identify pulmonary stenosis and regurgitation
Tricuspid valve	Free text analysis to identify tricuspid regurgitation. Impossible values of tricuspid regurgitation Vmax (≤0) were removed
RVSP	Direct numeric variable of RVSP prioritised as the final result. Otherwise, free text analysis would select the number preceding ‘mmHg’ as the RVSP. Erroneous values were manually corrected
Diastolic dysfunction	Diastolic dysfunction determined using criteria in guidelines.^12^
Right ventricular variables	Free text analysis to identify right ventricular size and function
Export dataset	Dummy variables and raw variables dropped from the dataset. Dataset sorted by hospital number and the earliest study date for each hospital number selected. Dataset was then exported

AoV, aortic valve; BMI, body mass index; IVSd, interventricular septum end diastole; LVEF, left ventricular ejection fraction; LVOT, left ventricular outflow tract; LVPWd, left ventricular posterior wall end diastole; NA, not available; RVSP, right ventricular systolic pressure; VTI, velocity–time integral.

#### Identification of repeat or focused studies

For the purposes of data cleaning, repeat/follow‐up studies on the same patient on a different date were not treated differently to isolated studies. Repeat studies on the same patient can be identified using the patient identifier and the study date. Focused studies with limited datasets were extracted but not specifically tagged in the data cleaning process, as accuracy was assessed against the individual elements of the clinical echocardiographic report, rather than comparison against a minimum dataset.

#### Numeric variables

Numeric variables were all handled in a similar fashion: impossible or extreme values were recoded as NA, and the variable was otherwise kept the same. The following variables were handled in a unique fashion.

##### Body mass index

Body mass index (BMI) was calculated from height and weight (rather than being in the report itself). Body mass index was calculated as the participant's weight in kilograms divided by their height (in metres) squared. Visual inspection of extreme BMI values revealed obvious instances where height and weight were transposed, and these were manually corrected (48 entries). A BMI ≥ 70 kg/m^2^ or ≤14 kg/m^2^ was considered too extreme and was recoded as NA.

##### Heart rate

Heart rate was often presented as a range of values and was recoded to be the mean of the two ends of the range. For instance, a heart rate of ‘120–180’ was recoded as 150 beats per minute (bpm). Heart rates over 200 bpm were excluded.

##### Right ventricular systolic pressure

For studies where the right ventricular systolic pressure (RVSP) numeric variable was entered, this value was used directly. Otherwise, the free text of the report was analysed, and the numerals preceding a ‘mmHg’ (the units of measurement of pressure) phrase were extracted. These were then coded as the RVSP. Entries were visually checked to ensure whether the value being coded was correct.

##### Left ventricular ejection fraction

A variety of techniques can be used to determine left ventricular ejection fraction (LVEF). Different methods were used in different patients, and sometimes, multiple methods were used in the same patient. As such, a hierarchy was generated that prioritised LVEF measurement in the following order: modified biplane, modified apical four chamber, modified apical two chamber, Teicholz 2D, M‐mode cube and M‐mode.

##### Removal of impossible values

For some variables, a negative value was not possible – for instance, LV posterior wall thickness cannot be zero or less. Therefore, for these variables, a value ≤0 was recoded as NA.

#### Categorical variables

Categorical variables were all handled in a similar fashion. The echocardiography report could contain either a true categorical variable (e.g. LV systolic function) or a free text section (e.g. left ventricle). In cases where the former was available, it was the preferred input. If not, then the free text section was analysed.

**Figure 1:**
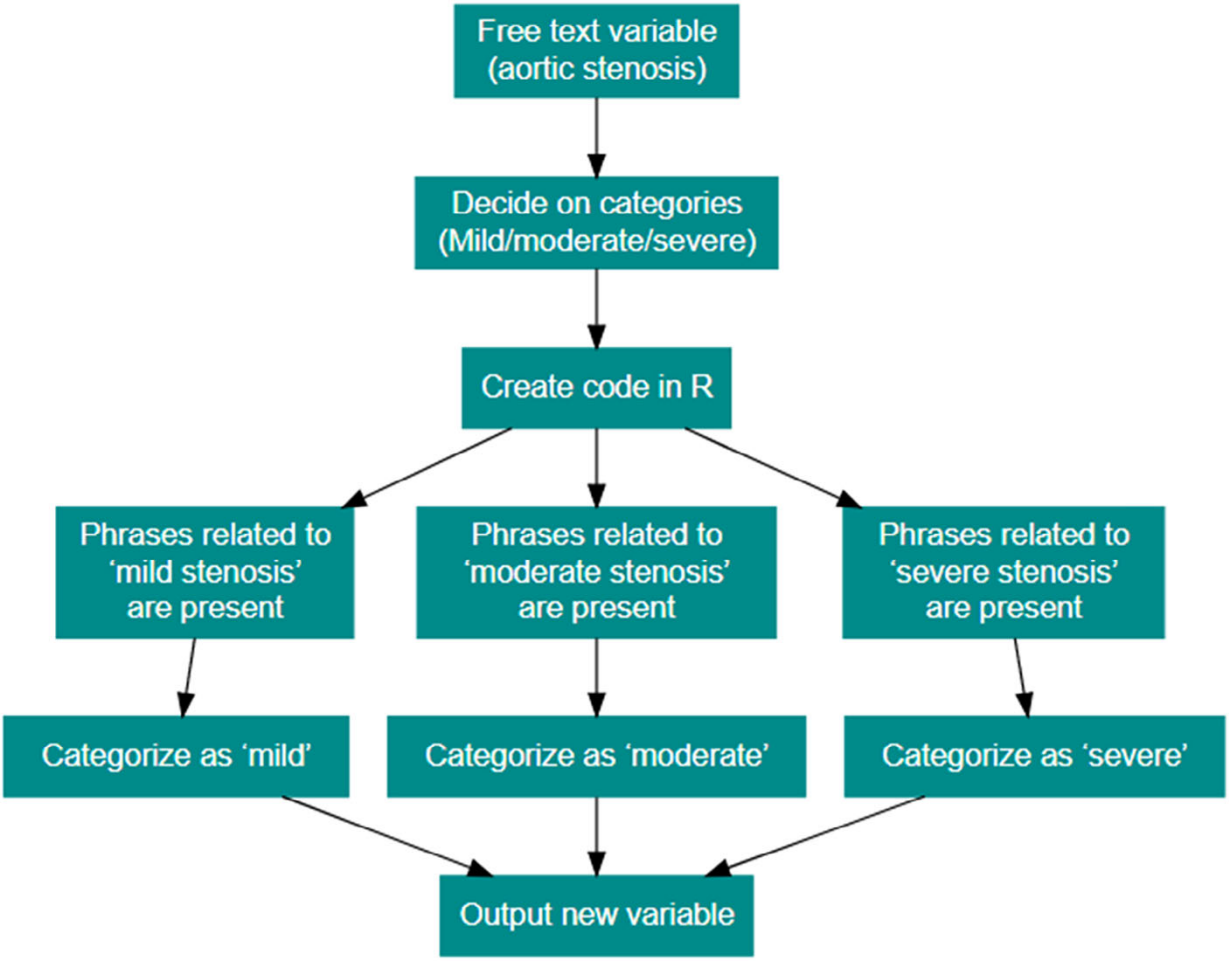
Categorisation of free text entries into categorical variables, using aortic stenosis as an example.

Free text is the major limitation to successful data analysis from a clinical database. However, frequently used phrases tend to become established in any echocardiography laboratory. Based on an initial set of phrases used commonly in local reports, a set of phrases were iteratively developed to detect free text entries that determined which level a categorical variable should fall into. An example of this process, using ‘aortic stenosis’ severity, is presented in Figure 1. Intermediate categories, in particular mild‐to‐moderate and moderate‐to‐severe classifications, were also assessed as a possible grading of a particular variable, via the same method as in Figure 1. This code was developed iteratively – that is, studies would be classified by the code and would be manually checked to see whether there were any obvious misclassification errors. If there were errors (such as typographic errors or alternative ways of reporting), these were corrected by editing the R code. Some examples of different phrases are given in Table 2.

**Table 2:** Examples of phrases used to describe left ventricular (LV) function.

Phrase	Classification
Systolic function appears within normal limits	Normal
LV function appears mildly and globally impaired	Mildly impaired
Systolic function is difficult to assess but visually appears mild‐to‐moderately impaired	Mild‐to‐moderately impaired
Moderate decrease in systolic function	Moderately impaired

#### Categorisation phrases

A selection of methods for certain variables is described below, because they were handled in a unique way or serve as a useful description for a method that was repeated for other variables.

##### Aortic stenosis

For full details of character terms in each category, please see the earlier linked R code. In brief, the following process was applied:

1. Whenever the word ‘thickened’ or ‘sclerosis’ is mentioned, ‘sclerosis’ was reported as the calcific aortic valve disease (CAVD) severity.
2. If any terms related to valve replacement (e.g. AVR, prosthesis and valve replacement) are mentioned, then the valve was assumed to be exogenous and was coded as an AVR.
3. Each free text entry was then searched for terms relating to CAVD severity. If a term denoting CAVD severity was reported, then it was coded as that.

##### Aortic regurgitation

Aortic regurgitation (AR) was extracted in a similar fashion to that of aortic stenosis. The only key difference is that additional ‘catch‐all’ string searches were included – for instance, if the word ‘moderate’ and ‘regurgitation’ appeared in the same sentence together, the entry would be categorised as ‘moderate regurgitation’. Importantly, this function does not misclassify AR due to the order in which the terms are evaluated (see R code). For example, one could conceive that if the sentence described ‘mild regurgitation’ and ‘moderate stenosis’, the catch‐all would interpret this as moderate regurgitation. This is not the case as the phrase ‘mild regurgitation’ would have already been categorised.

##### Aortic valve morphology

Aortic valve structure typically falls within one of the two categories – bicuspid or tricuspid. A very small portion of patients may have some other very rare morphology, and these studies were excluded due to very low numbers that would have likely limited patient anonymity. Entries containing the word ‘bicuspid’ were categorised as ‘bicuspid’. The remaining entries containing the word ‘tricuspid’, either stating the valve as normal or not stating the valve structure, were classified as ‘tricuspid’. This was done as if the valve structure was not clearly stated, the treating clinician would believe it to be tricuspid. A bicuspid valve is a clinically significant finding and is very unlikely to not be reported, if identified on the echocardiogram.

##### Diastolic dysfunction

We utilised the ASE/EACVI guidelines to establish whether participants had diastolic dysfunction.^12^ The decision tree is found in these guidelines, and a ‘normal LVEF’ for the two arms of the decision tree was defined as >50%.

##### Cardiac rhythm

Two separate variables were generated from the cardiac rhythm field in the report: a prioritised cardiac rhythm and the presence of bundle branch block (BBB). Bundle branch block was categorised as left BBB, right BBB, BBB (unspecified) or no BBB.

Prioritised cardiac rhythm could have the following values:

- artificially paced;
- supraventricular tachycardia;
- atrial flutter;
- atrial fibrillation;
- heart block;
- sinus rhythm;
- other; and
- uncertain.

##### Left atrial size

Where possible, as it was the clinically used value, the free text interpretation of the LA size was preferred. However, at times, only the measurement was available without a qualitative size given. In these cases, LA size was defined according to international guidelines.^13^

##### Remaining variables

All remaining free text variables (except for indications) were categorised in the same way: using the detection of phrases to place a study into the correct level.

##### Indications

A binary true/false variable was created for each indication category, and all participants were categorised as either fitting that indication (true) or not (false). A participant could have multiple indications. This level of detail was recorded to allow for more specific sub‐analyses, as well as the ability to collapse the categories down, if simpler analysis was preferred. The full list of indication categories is reported in the supplement (Table S2).

### Data validation

Given the iterative nature of the data cleaning, a data validation set was essential. A random set of 100 studies were selected, and the R code output was compared to the final echocardiography report uploaded on the participant's electronic health record. A Cohen's kappa was calculated for each variable in the dataset (all numerical variables matched). In all, this process was repeated three times, with the final validation set reported here. This is because discrepancies were identified in the first two sets, and issues were subsequently corrected. The assessment of indication categories was derived by calculating Cohen's kappa for *each indication category*. The average Cohen kappa across all indications is presented in Table 3.

**Table 3:** Final validation set of data miner output.

Variable	Proportion correctly matching clinical echocardiography report (%)	Cohen's kappa (κ)
Aortic valve stenosis	97	0.95
Aortic valve regurgitation	100	1.00
Aortic valve structure	98	0.96
Mitral valve stenosis	99	0.89
Mitral annular calcification	99	0.94
Mitral regurgitation	95	0.86
Tricuspid regurgitation	99	0.99
Pulmonary stenosis	100	1.00
Pulmonary regurgitation	98	0.96
LV size	92	0.74
LV function	93	0.89
LA size	100	1.00
RV size	97	0.89
RV function	97	0.91
Sex	98	0.96
Age	100	0.98
Indications	95	0.88^a^
Cardiac rhythm	99	0.98
Bundle branch block	100	1.00

LA, left atrium; LV, left ventricle; RV, right ventricle.

^a^ Mean value of all kappa statistics for each indication category.

### Ethical approval

Consultation with Māori was undertaken with the Ngāi Tahu Research Consultation Committee. This study received ethical approval from the Central Health and Disability Ethics Committee in New Zealand (ref.: 21/CEN/15). A waiver of informed consent was granted as part of this ethical approval. Locality approval was sought from and provided by the Southern District Health Board.

## Discussion

This study aimed to develop a dataset linking clinically used echocardiographic data to outcomes in New Zealand. We aimed to use these data to investigate the differences in survival and natural history of various conditions. We demonstrate excellent validity of our dataset, by comparing it against the clinically used electronic health record.

As mentioned in the background, recent work internationally has resulted in the use of several echocardiography databases to examine conditions of the cardiovascular system. The NEDA study, alongside databases from Israel and the United States, have examined outcomes in conditions such as moderate aortic stenosis, pulmonary hypertension, and mitral, tricuspid and aortic regurgitation.^3^, ^4^, ^5^, ^7^, ^8^ We aimed to further add to this literature using this generated dataset.

Our dataset has several strengths. First, validation of the output by comparison with the electronic health record means that we can be confident that the variables represented are accurate to the clinical information used by healthcare providers. Second, we were able to link patients to granular outcome data, including surgeries, allowing for the potential of more in‐depth analyses. Finally, because our data cleaning uses free, open‐source software, our methods can inform future techniques for other researchers who may be considering a similar type of analysis.

Our intention is to use these data to examine the natural history of early calcific aortic valve disease, and the epidemiology of other forms of valvular abnormalities in a New Zealand population. However, our approach could be used by other clinical or research teams on the databases powering their own PACSs, in order to answer clinical research questions or perform quality improvement studies. Many PACS vendors now offer data analytics tools, to which the methodology described could be readily applied or altered, and machine learning‐based large language models are likely to improve data extraction further. The PACS is more than a simple image repository: it contains great potential to answer clinically relevant research questions and thus improve our ultrasound practice. The challenge is accessing the data in an accurate and meaningful way.

## Author contributions

**Matthew K Moore:** Writing – original draft, and review & editing; formal analysis; investigation; funding acquisition; validation; methodology. **Gillian Whalley:** Writing – review & editing; methodology. **Gregory T Jones:** Conceptualization; supervision; writing – review & editing. **Sean Coffey:** Conceptualization; supervision; writing – review & editing.

## Funding

Matthew K Moore was generously supported by the National Heart Foundation of New Zealand and the E & W White Parsons Charitable Trust. Funding for the study was provided through a grant from the Department of Medicine, University of Otago.

## Conflict of interest

Gillian Whalley is an author of this manuscript and serves as Editor‐in‐Chief for the *Australasian Journal of Ultrasound in Medicine* and was excluded from the handling of and decison making related to this manuscript.

## Supporting information


**Table S1.** List of extracted variables.
**Table S2.** Indication categories.
